# Assessment of a Multifunctional River Using Fuzzy Comprehensive Evaluation Model in Xiaoqing River, Eastern China

**DOI:** 10.3390/ijerph191912264

**Published:** 2022-09-27

**Authors:** Yongfei Fu, Yuyu Liu, Shiguo Xu, Zhenghe Xu

**Affiliations:** 1 School of Water Conservancy and Environment, University of Jinan, Jinan 250024, China; 2 School of Hydraulic Engineering, Dalian University of Technology, Dalian 116024, China

**Keywords:** multifunctional river, indicator system, fuzzy comprehensive evaluation method, Xiaoqing River

## Abstract

Rivers are beneficial to humans due to their multiple functions. However, human meddling substantially degrades the functions of rivers and constitutes a threat to river health. Therefore, it is vital to assess and maintain river function. This study used the Xiaoqing River in Shandong Province, China, as a case study and established a multilayered multifunctional river evaluation indicator system consisting of environmental function, ecological function, social function, and economic function. The weights of indicators were calculated using the analytic hierarchy process (AHP) and the entropy method. Furthermore, a fuzzy comprehensive evaluation model based on the Cauchy distribution function was developed to assess the operation status of each function in each river segment. The results of the indicator and criterion layers in different river sections varied. The multifunctionality of the river decreased from upstream to downstream. The Jinan section was the most multifunctional, followed by the Binzhou, Zibo, and Dongying sections, and finally the Weifang section. Through additional analysis, this study determined the constraint indicators and functions of each river section. Overall, the results reveal that the idea of a “multifunctional river” can advance the theoretical understanding of a river’s function, and the fuzzy comprehensive evaluation model is demonstrated to provide fresh perspectives for evaluating river function.

## 1. Introduction

Rivers serve as the foundation of human society and culture, fostering social progress through natural functions and human civilization through the values of social, economic, and environmental wellbeing [1]. River exploitation activities have never stopped and are only intensifying [2,3]. Realizing the effective use of river functions is the primary goal of this paper. The disruption generated by human activity has violated the laws of change in the natural evolution of rivers and put river ecosystems under varying degrees of stress [4], causing many of the functions of rivers to deteriorate or even disappear. Therefore, a fair assessment of river functions is required in order to comprehend the current development of each function. This can serve as a foundation for river management and help to ensure that rivers are developed in a way that balances human activity and natural evolution [5].

River evaluations mostly focus on the advantages and disadvantages of rivers in their natural form, paying little regard to the river’s role as a resource for people [6,7]. People frequently ignore the opportunity that rivers offer for the sustainable development of human society in favor of human interventions aimed at protecting rivers, even when recognizing the socioeconomic significance of rivers. Wang et al. [8], Qishlaqi et al. [9], and Di et al. [10] analyzed the state of a river’s water environment using a variety of evaluation indicators. Wang et al. [11] established an assessment index system to assess river health in the Wei River Basin on the basis of the river’s natural function. Zhang et al. [7] constructed a watershed ecological security evaluation and proposed an ecological regulation approach for the East-Liao River basin based on the river’s ecological function. As is apparent, most of the studies mentioned above were based on a particular function of the river, with were few qualitative and quantitative studies on all functions. These studies frequently ignored the reality that river systems are complex ecosystems made up of subsystems, with a natural ecology and a social economy.

The reasonable assessment of each river function is the core component of multifunctional river evaluation. The multi-indicator evaluation method and the predictive model method are the two categories under which the methods of evaluating river function are classified [5]. By comparing the theoretical species status in the empirical model of the river to be evaluated with the actual species composition of the river, the predictive model method can show changes in river functions by presuming that all changes in a river are reflected in a selected species [12]. Typical predictive model methods are the River Invertebrate Prediction and Classification System (RIVPACS) [13], the Australian River Assessment Scheme (AusRivAS) [14], and Hydro-Geomorphic Assessment (HGM) [15]. This method is ideal for modeling a single ecological process or ecological function. It is difficult to explain the full ecological process and function of a river using this method. However, multifunctional river evaluation is multilevel and all-encompassing, taking into account both quantitative and qualitative factors. Therefore, a comprehensive evaluation method should be chosen. The multi-indicator evaluation method is appropriate for evaluating multifunctional rivers because it effectively characterizes the status of each river function through a judicious choice of evaluation indicators. Representative multi-indicator evaluation methods include the Index of Biological Integrity (IBI) [16], the Index of Stream Condition (ISC) [17], and the River Habitat Survey (RHS) [18]. A few scholars have recently conducted studies on the comprehensive examination of river functions. Xu et al. [19] divided river functional regionalization and categorized river functions as natural resources, human social activities, and ecological environment. By defining and describing river functions, Deng et al. [20] established an indicator system for river health assessment. To assess the coordination and growth of natural and social functions, Chen et al. [21] built a coordinated development degree model. They demonstrated how the evaluation of river function has evolved over time from a single assessment of river water quality, biological habitat, and water ecology to a whole evaluation based on all river functions.

Current studies on the evaluation of river function typically focus on continuous natural rivers and ignore rivers that are stopped by gates and dams or are subject to artificial management. However, there is room for further research on rivers affected by both natural and anthropogenic factors. Thus, the Xiaoqing River, which is in eastern China and is subject to multiple natural and human-caused pressures, served as our case study.

In Shandong Province, China, the Xiaoqing River is a prized waterway that may revive intermodal traffic between river and sea. The ecosystem of the river, however, has been severely harmed as a result of the discharge of home and industrial effluent, rendering the river impassable [22]. Recent years have seen steady promotion of the Xiaoqing River basin ecological environment comprehensive management plan and a progressive restoration of the watershed ecosystem [23]. The Xiaoqing River study is currently being conducted at the watershed and regional scales, and the evaluation findings are somewhat broad and challenging to apply to river management [24,25]. As a result, in order to determine the Xiaoqing River’s functional state as precisely as possible, this study established a multi-indicator and multilevel multifunctional river evaluation indicator system and assessed its function at the level of river segments. Additionally, we established a number of evaluation criteria. The indicator weights in this study were determined using the analytical hierarchy process (AHP) and the entropy method. The multifunctional river was evaluated using the fuzzy comprehensive assessment model.

This study makes advances in terms of theory, methodology, and its case study compared to previous studies on the functions of rivers. First, this study introduces the concept of a “multifunctional river”. This could advance the theoretical understanding of river functions. Second, the multifunctional river evaluation indicator system established in this study covers all functions of rivers and provides a comprehensive evaluation of river functions. Third, the fuzzy set theory was applied to multifunctional river evaluation and the Cauchy distribution function was applied as the affiliation function. This approach treats river functions systematically, allowing decisions to be made in a comprehensive way. The problem of quantifying a large number of uncertain factors in the evaluation was solved. Lastly, the Xiaoqing River selected for the case study is representative of a river affected by both natural and human pressures. As our evaluation was carried out at the level of river segments, the results can provide the management department’s decision-makers and scientific research institutions with accurate and unbiased information on the functional operation status and change trend analysis of the Xiaoqing River’s main channel, which can serve as a foundation for the river’s future functional improvement and ecological restoration.

## 2. Materials and Methods

### 2.1. Study Area Data

**Figure 1:**
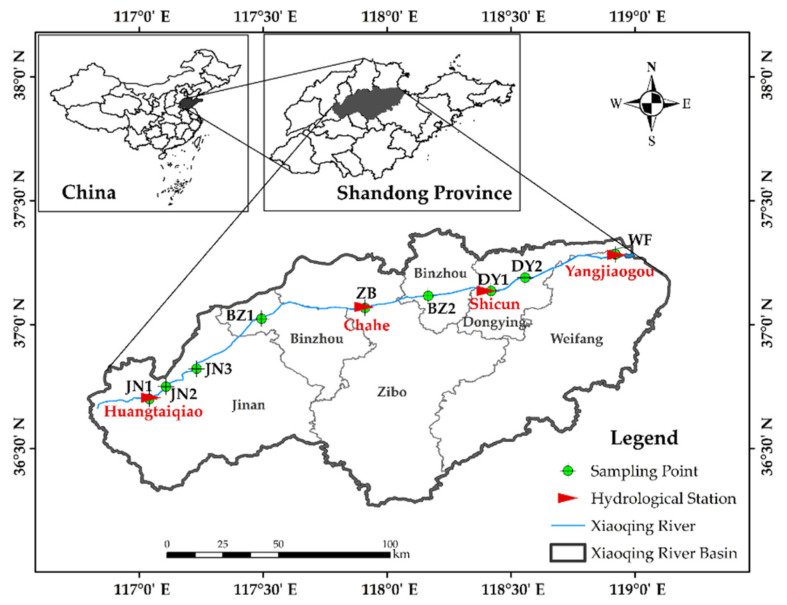
Map of Xiaoqing River basin.

The Xiaoqing River basin (116°50′–118°45′ E, 36°15′–37°20′ N) (Figure 1) is located in the southern part of the Lubei Plain, with a watershed area of 10,498.8 km^2^. With a length of 233 km, the Xiaoqing River flows through Jinan, Zibo, Binzhou, Dongying, and Weifang before emptying into Laizhou Bay east of Yangkou Town in Shouguang. The overexploitation of the river, severe pollution of the water body, deterioration of the water environment, endangered aquatic life, degradation of various functions, and total cutoff of the waterway occurred after the 1970s as a result of the rapid development of industries along the river and the massive discharge of pollutants [22]. In 2007, a comprehensive management project for the Xiaoqing River was established on the basis of the idea of sustainable development, and a comprehensive ecological and environmental management plan for the river basin was progressively advanced. The Xiaoqing River navigation restoration project was formally inaugurated in September 2017, and an extensive flood control project was put into action alongside it.

In this study, we divided the main stream of the Xiaoqing River into five sections for evaluation: the Jinan section, Binzhou section, Zibo section, Dongying section, and Weifang section. Furthermore, 2020 was chosen as the evaluation year. On the main stem of the Xiaoqing River, there are four hydrological stations: Huangtaiqiao, Chahe, Shicun, and Yangjiaogou. Monitoring information from hydrological stations within the boundaries of each administrative region was used to compile the hydrological and sediment data. These comprise two river segments in the Binzhou section, which keeps track of information from the Chahe hydrological station. The main stream Xiaoqing River has nine sampling points set up to check for river water quality, aquatic species, and sediment on the basis of the physical characteristics and hydrological circumstances of the waters. Table 1 displays the locations of each sampling site.

### 2.2. Indicator System

#### 2.2.1. Indicator System Framework

The secret to evaluation is creating a rational and scientific indicator system. The reliability of the results of multifunctional river evaluation is directly impacted by the scientific nature, logicality, and effectiveness of the indicators. The evaluation procedure is made simpler and the true status of river function can be obtained correctly by using the right evaluation indicators for river function. To achieve this, the multifunctional river evaluation indicators chosen must adhere to the fundamental scientific principles of systematization, representativeness, purposiveness, and uniformity. Additionally, because each river is different in terms of its natural properties, biological environment, and socioeconomic setting, each river serves a distinct function. As a result, each river should have a different set of multifunctional river evaluation indicators.

The frequency statistics method was chosen for the preliminary screening of indicators, and the primary indicators were further screened using the theoretical analysis method. The frequency statistics method is a method for counting the frequency of indicators that appear in articles connected to the evaluation of river function, then choosing the indicators that appear most frequently. In the indicator system, indicators that appeared more than ten times were chosen. The theoretical analysis method is a comprehensive analysis method that selects important indicators through theoretical analysis. We propose the concept of a multifunctional river in this study; after examining the meaning of the indicators and the connotation of each function of the river, we integrated the indications with overlapping connotations and further screened out the indicators that fit the criteria for each function. Furthermore, taking into account the potential for acquiring indicator data, we finalized the indicators according to the natural and social characteristics of the Xiaoqing River.

On the basis of the abovementioned methods, this study established a bottom-up multilayered multifunctional river evaluation indicator system with four levels (Table 2): the indicator layer, sub-criterion layer, criterion layer, and target layer. First, we determined that the target layer was a “multifunctional river”. River function, also known as river benefit, is the capacity and usefulness of the river system in its interaction with the environment [20]. Different effects are seen when river function depends on human requirements and when it depends the surrounding environment. Environmental function, ecological function, social function, and economic function are the main categories in this context. It is possible to coordinate and improve the many functions of multifunctional rivers in a balanced manner. In order to accomplish the ultimate aim of sustainable and coordinated development of people and rivers, it is possible to achieve a sustainable supply of river functions while guaranteeing the stability of the river’s structure and functions. As a result, we used the primary functions of the river as the foundation of the criterion layer in order to represent the characteristics and capabilities of the multifunctional Xiaoqing River from many angles. Second, the primary functions of a river can be subdivided into secondary functions. The sub-criterion layer of the evaluation system was made up of the secondary functions, which reflect how each function of the river is now being used and how it is always available. Lastly, to characterize the current status of each river function in relation to the various roles played by other river functions, a variety of indicators that directly reflect the current status of each river function were chosen for this study.

#### 2.2.2. Characterization of River Functions

(1) Environmental function. Environmental functions include hydrological function, water purification function, and self–repairing/regulation function. Rivers’ hydrological function refers to their contribution to the global atmospheric water cycle [26]. The monthly average flow rate of change (C1) and the degree of ecological flow satisfaction (C2) were used to characterize the hydrological function. Water ecosystem and river hydrological connectivity change as a result of changes in river flow [27]. C1 represents the geometric mean of the rate of change of the river’s mean monthly flow. Ecological flow is the bare minimum flow needed to preserve ecological balance, pollution dilution self–purification, river water–sand balance, and aquatic life presence [28]. C2 is the percentage of all days where the river’s observed daily average flow is larger than its ecological flow throughout the assessment year [29]. The river’s ability to eliminate pollution within its pollution carrying capacity is its water purification function [30]. This study used the rate of drinking water source water quality standards (C3), surface water quality (C4), and substrate contamination index (C5) to characterize the water quality purification function. River water quality affects the river ecosystem and is a key indicator of the river’s environment [31]. C3 refers to the ratio of drinking water sources that meet water quality criteria. We used a single factor water quality mark method to calculate C4′s sample data according to the “Surface Water Environmental Quality Criteria” (GB3838-2002, China). C5 can estimate the ecological danger of heavy metals in river silt [32]. C5 is the maximum pollutant multiplier value over the standard concentration in sediment. “Soil Environmental Quality Soil Contamination Risk Control Standards for Agricultural Land” (GB15618-2018, China) provided the pollutant concentration values. The self–repair/regulation function means a river’s ability to regulate internal changes and resist external shocks. This study used dissolved oxygen concentration in the water column to estimate self–purification capacity (C6) [33]. A faster rate of dissolved oxygen in a water body returning to its starting state indicates greater self-purification ability [34].

(2) Ecological function. Biological habitat function, river corridor function, and soil and water conservation function are the main ecological functions. The biological habitat function of rivers entails that they provide a living environment for ecosystem creatures [35]. Many factors affect habitat function. This study focused on species survival and biodiversity. Fixed, short–lived, and susceptible to pollutants, plankton habitats can reflect the disturbance and pollution of water bodies [11]. Therefore, plankton diversity was utilized to assess river habitat quality [36]. Benthic macroinvertebrates are aquatic invertebrates that may be seen with the unaided eye on the river’s bottom or adhering to aquatic plants and rocks. They can serve as indicators of biological habitat status because their species and community structure are very closely tied to the caliber of the immediate environment [37]. The biological habitat function was characterized using phytoplankton, zooplankton, and benthic macroinvertebrate diversity indices. The phytoplankton, zooplankton, and benthic macroinvertebrate Shannon–Wiener indices were calculated [38]. The river corridor function implies rivers’ linking of material, energy, and information between waterways and other ecosystems [39]. The river vertical connection index (C10) and landscape fragmentation (C11) were utilized to characterize this function. The longitudinal connectedness of river material cycles, information exchange, and biological migration influences the regional and temporal distribution of water quality and natural community species [40]. C10 measures a river’s longitudinal connectedness as a function of the number of artificial structures per 100 km of river [41]. Human meddling changes the river corridor scenery from a single homogeneous and continuous whole to a complex and diverse discontinuous mosaic [42]. C11 can characterize river corridor fragmentation and geographical complexity [43]. Fragstats were used to calculate and score the fragmentation index. The soil and water conservation function refers to how river water affects the erosion, transport, and deposition processes of topsoil and surface rocks [44]. This function was described using suspended sand transportation modules (C12). The severity of the watershed’s erosion increases with a greater sand transport modulus [45]. C12 is the ratio of transported suspended material to the watershed catchment area over a specific time period.

(3) Social function. Social function includes the flood control and transportation function, water supply function, and recreational function. The flood control and transportation function refers to rivers’ roles in flood relief and inland river transportation. The flood control function was characterized by the attainment rate of flood control engineering measures (C13). FT1 is the ratio of river flood barrier length to river embankment length. C13 evaluates a river’s flood-control capacity [27]. This study employed the percentage of navigable river sections (C14) to characterize river transport function. More navigable rivers have a better transport function [46], showing that the river can produce fresh water resources. This study employed the modulus of groundwater resources (C15) and the utilization rate of water resource (C16) to characterize the water supply function. C15 refers to subsurface water sources per area. The size of C15 depends on hydrogeological conditions, precipitation, and other elements, which can indicate the river’s water supply capability [47]. C16 is the ratio of basin surface water supply to surface water resources, which can characterize the river’s water resource development and use in the current year [27]. The river’s recreational function is that it offers users a water–friendly recreational area [48]. The degree of human activity demand satisfaction (C17) was employed in this study to describe this function. C17 refers to riparian residents or tourists’ satisfaction with landscape, recreation, and other facilities.

(4) Economic function. The economic function includes the economic benefit, aquaculture benefit, and tourist industry benefit. This is a reference to the river’s capacity to generate economic benefit for the community. The per capita Gross Domestic Product (GDP) (C18) and the water consumption of CNY 10,000 GDP (C19) were used to characterize the economic benefit. C19 refers to the ratio of total yearly water use to GDP in the river’s watershed. The macroeconomic performance of a region can be understood and grasped using C18 and C19, which are frequently employed as measures of economic development [49,50]. This study characterized aquaculture benefit using fish production capacity (C20). We calculated the status quo by counting and valuing the river’s fish species. The average tourist flow index (C21) and the visibility of scenic area (C22) were determined to characterize the tourist industry benefit. The daily ratio of tourists entering the riverine scenic area to the basic daily visitor number is known as the tourist flow index. To calculate the size of C21, we took the average value of the tourist flow index over the course of a year. The popularity of the scenic area may be a reflection of the publicity and promotion efforts made by the area, which have a subsequent impact on the benefits of tourism [51]. By conducting research and speaking with the appropriate tourism agencies, we were able to gather this information.

### 2.3. Evaluation Standards

The aims of riverine function assessment vary widely due to variations in geography, climate, and economics, making it challenging to standardize the indicators and scoring criteria [52]. The scoring criteria for riverine function indicators based on the attributes of the watershed environment were determined in this article using the critical threshold and expert consultation methodologies. The critical threshold method takes into account both social pressure and human involvement while viewing the river environment in its unaltered state as the ideal state. The ideal value is the initial state without any interference. The score then drops as the level of disruption rises. Moreover, if pertinent state departments had recently issued technical guidelines or industry standards, these were be preferred. For instance, the Technical Guidelines for River and Lake Health Assessment (SL/T 793-2020, China) were used to determine C2.

Lastly, this study divided the standard values into five grades: I (excellent), II (good), III (moderate), IV (bad), and V (poor) (Table 3) for reference to related studies.

### 2.4. Fuzzy Comprehensive Evaluation Model

In order to express uncertainty, in 1965 the American automatic control expert Zadeh developed fuzzy set theory [53]. The fuzzy comprehensive evaluation method is a comprehensive evaluation approach built on fuzzy set theory. Using an affiliation degree to describe fuzzy boundaries converts qualitative evaluation into quantitative evaluation. This method separates the estimated change interval of a target and thoroughly assesses the status of the attachment level using a number of indicators. On the one hand, it considers the target’s hierarchical structure to reflect the fuzziness of the evaluation standards and affecting variables. On the other hand, it fully incorporates human experience into the review, improving objectivity and aligning the evaluation results with the actual situation [54]. The approach combines qualitative and quantitative elements with distinct and organized impacts, and can effectively address a wide range of problems that are otherwise challenging to measure.

#### 2.4.1. Constructing the Weight Vector

Weighting is commonly used to quantify the relative importance of different things [46]. Subjective and objective procedures can be used to apply indicator weights. Although the AHP’s operation is rather straightforward, it is arbitrary and subjective [19]. The entropy technique can account for the impact of the indicator’s current value on the evaluation outcomes, which is more in accordance with objective reality [55]. In this study, weights were determined using the AHP and entropy methods, and total weighting values were then determined using the weighted average concept. This method can make evaluation more precise while lowering randomness.

1. The Analytic Hierarchy Process (AHP)
  (1) Construct the judgment matrices. Each function in the criterion level and the relationships between each indicator in the indicator level are included in the judgement matrix. We used the 1–9 scale approach to rate the relative value of *n* elements within the same story.
  (2) Calculate the weight vector and determine test consistency.
  (3) Calculate the combined indicator-to-target weight vector and execute the combined consistency test. The formulas are as follows:
    (1) $$\lambda_{\max}=\overset{n}{\underset{k=1}{\sum }}\frac{{(A\alpha }_{k})}{{n\alpha }_{k}},$$
    (2) $$C.I.=\frac{\lambda_{\max}-n}{n\;-1},$$
    (3) $$C.R.=\frac{C.I.}{R.I.},$$
    where $\lambda_{\max}$ is the maximum characteristic root, A is the judgment matrix, $\alpha_{k}$ is the weight vector, and R.I. is the stochastic consistency indicator of the judgment matrix. When C.R. < 0.1, the judgment matrix is considered to be acceptable.

2. The Entropy Method
  (1) Construct the base matrices. A base matrix contains each indicator’s real values at different selection points:
    (4) $${X=(x}_{\mathrm{ij}}{)}_{m\times n}$$
    where m and n are the number of rows and columns in the matrix, respectively, and $x_{\mathrm{ij}}$ denotes the initial values of the *j*th indicator at the *i*th selection point.
  (2) Normalization process. The normalization formulas for positive and negative indicators are different. The formulas are provided below.

When the indicator is positive:

(5) $$r_{\mathrm{ij}}=\frac{x_{\mathrm{ij}}-x_{\min}}{x_{\max}-{\;x}_{\min}},$$

When the indicator is negative:

(6) $$r_{\mathrm{ij}}=\frac{x_{\max}-x_{\mathrm{ij}}}{x_{\max}-x_{\min}},$$

where $x_{\min}$ is the minimum value of $x_{\mathrm{ij}}$, and $x_{\max}$ is the maximum value of $x_{\mathrm{ij}}$.

(3) Calculate entropy value. The amount of dispersion of an indicator was calculated using the entropy value. When an indicator’s information entropy value is low, it means that the distribution is significant and has a large impact on the evaluation as a whole [55]. The formulas are as follows:
  (7) $$f_{\mathrm{ij}}=\frac{{1+r}_{\mathrm{ij}}}{\sum_{j=1}^{n}{(1+r}_{\mathrm{ij}})},$$
  (8) $$H_{i}^{\prime }=\frac{\sum_{\;j=1}^{n}f_{\mathrm{ij}}{\ln f}_{\mathrm{ij}}}{\ln n},$$
  where $f_{\mathrm{ij}}$ is the proportion of indicator *i* in object *j*, $H_{i}^{\prime }$ is the entropy value of indicator *I*, and *n* is the total number of river sections.

(4) Calculate weights. The weighting formula is as follows:
  (9) $$\omega_{i}=\frac{1-H_{i}^{\prime }}{m-\sum_{i=1}^{m}H_{i}^{\prime }},$$
  where *m* is the number of indicators, $\omega_{i}$ is the weight of indicator *i*, 0 ≤ $\omega_{i}$ ≤ 1, and $\overset{m}{\underset{i=1}{\sum }}\omega_{i}=1$.

#### 2.4.2. Calculate the Affiliation Matrix

First, in order to determine the judgement sets representing the five states of specific evaluation indicators, namely, excellent, good, moderate, bad, and poor, we established the evaluation indicator sets of the target layer and each criterion layer. These sets correspond to the five levels of I, II, III, IV, and V in Table 3. The fundamental formula of the affiliation matrix was then calculated using the Cauchy distribution function as the membership function [56]:

(10) $$r(X)=\frac{1}{{[1+a}_{2}{(x-a_{1})}^{2}]},$$

where r(X) is the membership function, *x* is the current value of indicator *x* for the river segment to be assessed, and $a_{1}$ and $a_{2}$ are the function parameters.

When *x* is level I:

(11) $$a_{1}{=x}_{u},{\;a}_{2}=\frac{4}{{{(x}_{u}-x_{v})}^{2}},$$

When *x* is level II–IV:

(12) $$a_{1}=\frac{x_{u}{+x}_{v}}{2},{\;a}_{2}=\frac{4}{{{(x}_{u}-x_{v})}^{2}},$$

When *x* is level V:

(13) $$a_{1}{=x}_{v},{\;a}_{2}=\frac{4}{{{(x}_{u}-x_{v})}^{2}},$$

where $x_{u}$ and $x_{v}$ are the upper and lower boundary values of *x* corresponding to different levels of standard values, respectively.

Then, the affiliation matrix *R* was fuzzy multiplied with the weight vector *W* to yield the comprehensive evaluation vector *D*:

(14) D=W·R

Lastly, according to the principle of maximum affiliation, we determined the rank status of the target layer and obtained the final results.

## 3. Result

### 3.1. Comprehensive Weight

This study used MATLAB to compute the weight values of each indicator in two different ways, that is, in accordance with the AHP and entropy methods. The total weight was determined using the weighted average approach (Table 4). The environmental function had the highest weight in the criterion layer (0.301), whereas the economic function had the lowest weight (0.1964). Each indicator’s weight in relation to the target layer varied substantially, ranging from 0.0286 to 0.0763. The monthly average flow rate of change (C1) and self-purification capacity of water bodies (C6) were the environmental indicators that obtained the highest weight values. Fish production capacity (C20) and visibility of scenic area (C22) obtained the lowest weights. Overall, the results were most influenced by the river’s environmental function, followed by its ecological function, and the least by its economic function.

### 3.2. Multifunctional River Evaluation Results

#### 3.2.1. Indicator Layer

**Figure 2:**
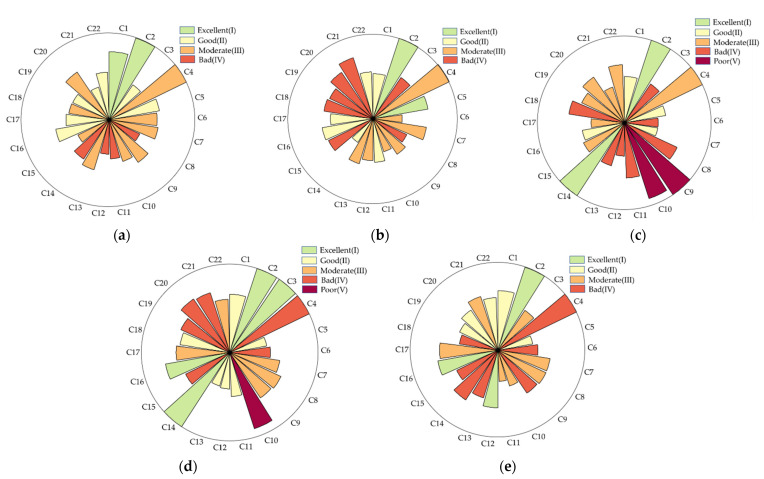
Indicator layer evaluation results. The results were obtained according to the principle of maximum affiliation. The size of the affiliation of each indicator with the rank is depicted in the figure. The maximum affiliation degree is 1. (**a**) Jinan section; (**b**) Binzhou section; (**c**) Zibo section; (**d**) Dongying section; (**e**) Weifang section.

In this study, a fuzzy comprehensive evaluation model was used to evaluate the Xiaoqing River; the results of the indicator layer evaluation are shown in Figure 2. Figure 2 depicts the affiliation of the indicator layer for each river section. The indications more closely reflecting the evaluation result level have a higher degree of affiliation. All river sections had excellent C2 values, which showed that the amount of water in the Xiaoqing River could satisfy the river’s ecological water needs. The Jinan section’s result was superior to that of the other sections, and the indicator values were primarily in the intermediate condition. Ecological and economic indicators in the Binzhou section were poor, with C3, C15, C18, C19, C20, and C21 as constraint indicators. The Zibo and Dongying sections had poor C10 values, indicating that the river connectivity is inadequate and there are more river gates and dams. The Zibo section’s constraint indicators were C8, C9, C10, C11, and C18. The Dongying section’s limitation indicators were C4, C10, C20, and C21. Notable is the fact that the Dongying section had the greatest number of indicators with excellent evaluation findings, albeit with a more discontinuous distribution. Ecological indicators in the Weifang section produced poor results; the constraint indicators were C4, C9, C13, and C14.

#### 3.2.2. Criterion Layer

**Figure 3:**
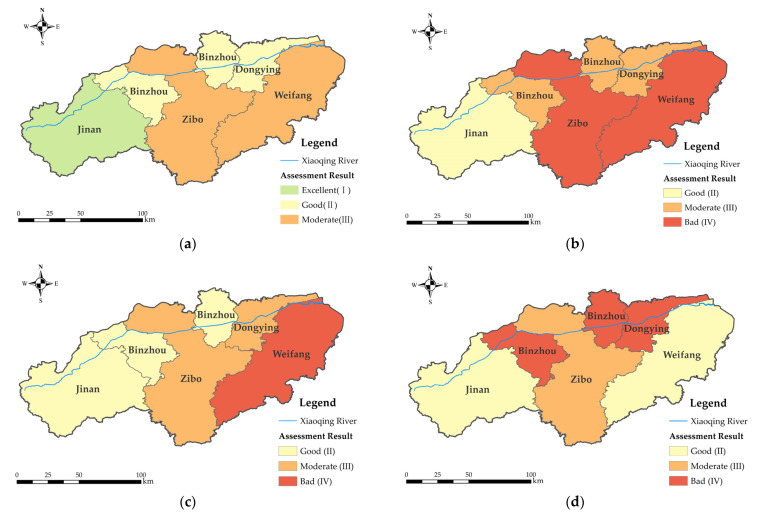
Criterion layer evaluation results: (**a**) environmental function; (**b**) ecological function; (**c**) social function; (**d**) economic function.

The results of the criterion layer in the study area are shown in Figure 3. The results of the upstream river sections were excellent, and the environmental function of the main stream of the Xiaoqing River was generally satisfactory. Overall, the river’s environmental function gradually declined from upstream to downstream, while the Zibo section’s evaluation level in the intermediate reaches was only moderate. Only the upstream part of the Jinan section was rated as having good ecological function, and evaluation results for the Zibo and Weifang sections were bad. The ecological function status of the main stream of the Xiaoqing River was unsatisfactory, which is consistent with its spatial fluctuation characteristics and environmental functions. The Jinan and Zibo sections, which are upstream, had better evaluation results in terms of social function, while Weifang, which is downstream, had worse evaluation results. The economic function was spatially heterogeneous. Better evaluation results were obtained from the river’s upstream and downstream sections. The Jinan and Weifang sections produced excellent results, and the Zibo section produced medium results, while the Binzhou and Dongying sections produced bad results.

#### 3.2.3. Target Layer

**Figure 4:**
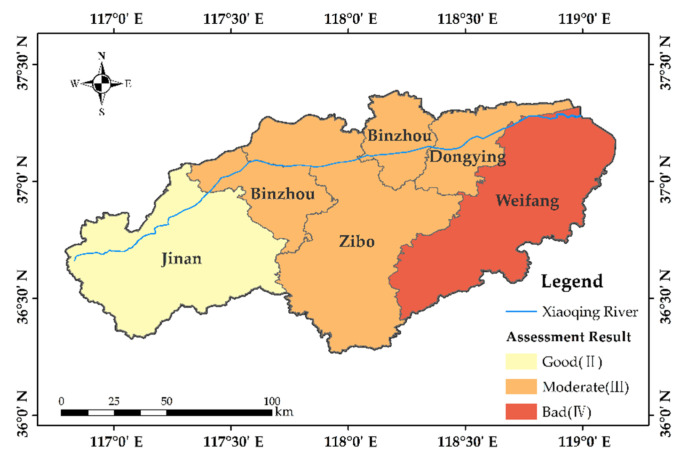
Target layer evaluation results.

Figure 4 displays the final evaluation results. Overall, the Xiaoqing River’s main stream had a moderate level of multifunctionality. The multifunctionality of the river decreased from upstream to downstream. The upstream section had the highest multifunctionality, and the results were good. This demonstrates that the functions could consistently and steadily supply human requirements, evolving in a coordinated and balanced manner. The middle sections of the river received a medium rating, indicating that while certain service functions were on the decline, they were still able to support human social development overall. The economic function represented the constraint criterion of the Binzhou and Dongying sections. The lowest multifunctionality of the downstream Xiaoqing River indicates that several of its functions are compromised and unable to support the needs of human social development. The ecological function and social function comprised the Weifang section’s constraint criteria.

## 4. Discussion

### 4.1. Establishment of Indicator System

There are numerous studies on how to assess the health of rivers. Typically, researchers select pertinent indicators to assess the condition of rivers [57]. However, quantitative assessments of river functions are rare in the literature. On the one hand, this is due to the complexity of the factors that affect how rivers function and the absence of clear classification criteria, which causes uncertainty in the evaluation objectives. On the other hand, limited economic resources and technological capabilities make it challenging to collect data for many variables. The fact that river function is the foundation of river health, however, cannot be disputed [21]. Therefore, river function evaluation indicators can objectively describe river health status. Chen et al. [21] used the same two indicators, C1 and C2, to assess river hydrological function. Deng et al. [20] chose several indicators to characterize river functions. In terms of the function of natural properties, the benthic macroinvertebrate index (H_bm_) and C9 have the same meaning. To symbolize the river’s ability to supply water, we unanimously settled on the utilization rate of water resources (C16). When evaluating the river health in the Luanhe River, Shan et al. [27] used C16. Meanwhile, Shan et al. confirmed the rate of drinking water source water quality standards (C3) as the evaluation indicator of water quality. Both C16 and C3 indicators were chosen by Xue et al. [58] in their investigation.

In summary, researchers usually use the same typical indicators to assess river water quality and water resource conditions in general. However, generally only one type of aquatic organism was used to evaluate the ecological function of rivers, which is not representative, when indicators were established for the overall function evaluation of rivers. The difficulties in monitoring aquatic species and the limitations of data collection may be the cause of this. The advantage of this study is that aquatic organisms in the Xiaoqing River, including phytoplankton, zooplankton, and macrobenthos, were thoroughly investigated. As a result, the results of our ecological function evaluation are more precise and trustworthy. Additionally, past research has often ignored the economic benefits of river functions. Economic advantages can clearly show the value that river functions generate. Economic functions were added to the criterion layer in this study, making the evaluation system more comprehensive and valuable.

### 4.2. Analysis of Evaluation Results

The Xiaoqing River’s upper reaches had surface water quality that met the III level, while the river’s lower reaches were still at the IV or V level, according to water quality sampling data. During the dry season, the downstream river experienced organic pollution and eutrophication issues; the main pollutants were total phosphorus, ammonia nitrogen, and permanganate. The main stream of the Xiaoqing River had a low dissolved oxygen concentration that fluctuated from 2.72 to 3.59, and the self-purification capacity of the water body (C6) was subpar. The water quality of the Xiaoqing River in Jinan improved as a result of treatment. The river’s downstream region, which has a great deal of farmland and many buildings built without permits, is a significant grain-producing region. Because of the ease with which pollutants are transported from farms to the main stream through ditches and tributaries, the water quality was impacted. Farmland surface source pollution and residential sewage discharge from rural residents were the main contributors to high pollutant levels. Furthermore, fish farming is practiced in the Weifang section, and, during the process of exchanging water for farming, leftover bait and manure can enter the river, producing pollution. The habitat of aquatic species was obliterated as a result of the long-term contamination of the river’s water quality. The habitat of aquatic species and the landscape along the river would have been somewhat disturbed by project construction [42,59]. The water biological assessment of the Xiaoqing River revealed a total of 49 species of phytoplankton, according to the sampling data. The phylum of green algae accounted for the most species, making up 41% of the total. Eleven different zooplankton species in all were studied. The swimming suborder accounted for the most species, with six different varieties. The sampling results showed that the phytoplankton diversity index (C7) ranged from 1.38 to 1.92 and the zooplankton diversity index (C8) varied from 0.69 to 1.58 for the mainstream Xiaoqing River, both of which were at a low level. A total of thirteen benthic macroinvertebrates species were discovered. Because there was only one species present, the Shannon–Wiener index at the Chahe hydrological station was 0. Overall, the Xiaoqing River’s aquatic organism diversity index and extant species rate were low. The main Xiaoqing River was poorly connected, as it is a manmade river with many gates and dams. The Zibo and Dongying sections are where this is most visible. The Jinan section’s landscape fragmentation (C11) reached 81.2, with engineering development causing additional harm to the river’s landscape.

Although multiple treatment measures have substantially increased the Xiaoqing River’s capacity to withstand flooding, floods started occurring annually in the Xiaoqing River watershed as of 2018. Therefore, it is necessary to enhance the river’s flood control capabilities. There are numerous industrial plants and dense population centers along the Xiaoqing River; hence, there is a high need for water resources. As a result, the Xiaoqing River basin is experiencing major groundwater overdraft, and the use of water resources is increasing. The Xiaoqing River Navigation Restoration Project’s implementation in recent years has encouraged the integration and classification of tourism resources as well as economic growth along the river. Examples include the ancient city of Qingzhou in Weifang, the Matanhu National Wetland Park in Zibo, and the Xiaoqing River Source Wetland Park in Jinan. Development of the Xiaoqing River basin’s tourism resources may bring large economic benefits along with major ecological and social advantages.

In general, the results of our multifunctional river evaluation mostly agreed with the river’s actual state. The findings provided information on the state of development of each function of the Xiaoqing River, which could be used to direct restoration efforts for those functions in need of attention and increase efforts to protect those in better condition. The Xiaoqing River’s functions were found to be operating poorly in several areas due to heavy human intervention.

### 4.3. Further Research Perspectives of the Current Study

This study used a hierarchical progression to establish an indicator system for evaluating river function, with the Xiaoqing River as a case study. The intersection of many water networks, which is a “natural–artificial” composite water network system, is where the Xiaoqing River originates. Both natural and human activities have an impact on how the river functions. Therefore, the multifunctional river evaluation indicator system suggested in this study can be used with rivers of the same type, with results that could inform river management and ecological restoration. However, this study did have limitations. We could not entirely cover the entire research region with the few sampling points we were able to choose. Furthermore, because of the limitations of the underlying data, the indicators used in this study were unable to fully represent all of the functions of the river. Researchers should, therefore, continue to refine the evaluation system using more representative indicators for subsequent investigations.

In recent decades, the study of river function has gradually grown and evolved. There are numerous issues with the available research on the overall function and assessment of river systems, even though the evaluation of river ecosystem function and certain specific functions of rivers are reasonably well developed compared to earlier evaluations. As a result, research on river function can be improved and examined from the following angles in the future:

(1) Extend and refine the fundamental theory and notion of river systems and functions;
(2) Create a collection of practical and scientific methodologies for assessing river function;
(3) Establish a fair definition of the requirements for using the evaluation method;
(4) Integrate river development, utilization, and management with the evaluation outcomes.

It is important to note that during our evaluation of ecological function we discovered that the environmental assessment of rivers is primarily based on water quality evaluation, which to a certain extent ignores the impact of hydrology, water quality conditions, and geomorphological changes on rivers’ biological communities [57,60,61]. As a result, it would be wise to construct a monitoring system for aquatic creatures in important rivers. To accurately grasp the trends of ecological water changes in important waters and gather monitoring data for future research on river function and river health evaluation, a long-term and continuous observation and survey program should be developed to conduct long-term surveys and monitoring of plankton, benthic animals, fish, and fishery resources.

## 5. Conclusions

(1) The concept of multifunctional rivers is gaining clarity. Multifunctional rivers’ multiple functions can complement one another and grow in a positive way. In order to accomplish the ultimate aim of sustainable and coordinated development of people and rivers, it is necessary to achieve a sustainable supply of river functions while protecting the stability of the river’s own structure and functions. The creation of multifunctional rivers offers a path towards achieving the coexistence of people and rivers.
(2) Our evaluation of the Xiaoqing River’s functions at the level of individual river segments can provide river management agencies with precise information on the state of various parts of the river, making it easier to create more targeted management plans and ecological restoration initiatives. The multifunctional river assessment indicator system, which was developed on the basis of the river’s four primary functions and twelve secondary functions, can thoroughly assess each function of the Xiaoqing River. The combination of the AHP and the entropy method can balance subjective and objective data. The development status of each function of the Xiaoqing River can be objectively represented by the fuzzy comprehensive evaluation model built using the Cauchy distribution function as the affiliation function.
(3) The overall multifunctionality of the mainstream Xiaoqing River was at a medium level. From upstream to downstream, the river’s multifunctionality decreased, with the upstream part having the maximum multifunctionality. Overall, the ecological function evaluation result was bad, and the Xiaoqing River had the best operational state for environmental function. The evaluation results can guide the Xiaoqing River’s future function enhancement and ecological restoration. The upstream stretch of the Xiaoqing River was in good functional condition and contained less pollution. Therefore, it is suggested that protective measures be taken for the upper reaches of the Xiaoqing River, and that oversight and management be strengthened. Several functions of the middle and lower sections of the Xiaoqing River were impaired, and remediation actions are required to restore the river’s multifunctionality. As the Xiaoqing River renavigation project progresses, the river habitat restoration project should be implemented across the entire Xiaoqing River basin. Xiaoqing River habitat restoration can be accomplished by splitting the river into functional zones. This is should be one of the main Xiaoqing River research goals for the future.

## Figures and Tables

**Table 1:** The sampling point location in the main stream of the Xiaoqing River.

Number	Section	Longitude and Latitude	Item
JN1	Jinan section	36.701° N, 117.041° E	Water quality, aquatic organisms, sediment
JN2	Jinan section	36.750° N, 117.108° E	Water quality, sediment
JN3	Jinan section	36.823° N, 117.230° E	Water quality, sediment
BZ1	Binzhou section	37.024° N, 117.493° E	Water quality, aquatic organisms, sediment
ZB	Zibo section	36.068° N, 117.911° E	Water quality, aquatic organisms, sediment
BZ2	Binzhou section	37.116° N, 118.166° E	Water quality, aquatic organisms
DY1	Dongying section	37.136° N, 118.420° E	Water quality, aquatic organisms, sediment
DY2	Dongying section	37.189° N, 118.556° E	Water quality
WF	Weifang section	37.275° N, 118.925° E	Water quality, aquatic organisms, sediment

**Table 2:** The list of indicators used for multifunctional river assessment.

Target Layer	Criterion Layer	Sub-Criterion Layer	Indicator Layer	Data Source
Multifunctional river	Environmental function (A1)	Hydrological function (B1)	The monthly average flow rate of change (C1)	Hydrological station monitoring data
			The degree of ecological flow satisfaction (C2)	
		Water quality purification function (B2)	The rate of drinking water source water quality standards (C3)	Water Resources Bulletin
			Surface water quality (C4)	Sampling data
			Substrate contamination index (C5)	
		Self-repairing/regulating function (B3)	Self-purification capacity of water bodies (C6)	
	Ecological function (A2)	Biological habitat function (B4)	Phytoplankton diversity index (C7)	
			Zooplankton diversity index (C8)	
			Benthic macroinvertebrate diversity index (C9)	
		River corridor function(B5)	River vertical connection index (C10)	Remote sensing
			Landscape fragmentation (C11)	
		Soil and water conservation function (B6)	Suspended sand transportation modules (C12)	Hydrological station monitoring data
	Social function (A3)	Flood control and transportation function (B7)	The attainment rate of flood control engineering measures (C13)	Water conservancy departments
			The percentage of navigable river sections (C14)	
		Water supply function (B8)	Modulus of groundwater resources (C15)	Water Resources Bulletin
			The utilization rate of water resource (C16)	
		Recreational function (B9)	The degree of human activity demand satisfaction (C17)	Questionnaire
	Economic function (A4)	Economic benefit (B10)	The per capita Gross Domestic Product (GDP) (C18)	Statistical Yearbook of Shandong Province
			The water consumption of 10,000 CNY GDP (C19)	
		Aquaculture benefit (B11)	Fish production capacity (C20)	Water conservancy departments
		Tourist industry benefit (B12)	Average tourist flow index (C21)	Statistical Yearbook of Shandong Province
			The visibility of scenic area (C22)	Questionnaire

**Table 3:** The classification criteria of multifunctional river assessment indicators.

Indicator	Unit of Measure	Indicator Direction	Criteria				
			Excellent (I)	Good (II)	Moderate (III)	Bad (IV)	Poor (V)
C1	%	−	[0, 0.2]	(0.2, 0.4]	(0.4, 0.6]	(0.6, 0.8]	>0.8
C2	%	+	[98, 100]	[90, 98)	[80, 90)	[60, 80)	[0, 60)
C3	%	+	[90, 100]	[80, 90)	[70, 80)	[60, 70)	[0, 60)
C4	-	+	5	4	3	2	1
C5	-	−	[0, 1)	[1, 2)	[2, 3)	[3, 5)	>5
C6	mg/L	+	>7.5	(5, 7.5]	(3, 5]	(2, 3]	[0, 2]
C7	-	+	>3	(2, 3]	(1, 2]	(0, 1]	0
C8	-	+	>3	(2, 3]	(1, 2]	(0, 1]	0
C9	-	+	>3	(2, 3]	(1, 2]	(0, 1]	0
C10	pcs/100 km	−	[0, 0.3)	[0.3, 0.5)	[0.5, 0.8)	[0.8, 1.2)	≥1.2
C11	%	−	[0, 30]	(30, 60]	(60, 80]	(80, 90]	(90, 100]
C12	t/km^2^	−	[0, 50]	(50, 100]	(100, 200]	(200, 500]	>500
C13	%	+	[95, 100]	[85, 95)	[70, 85)	[50, 70)	[0, 50)
C14	%	+	[90, 100]	[80, 90)	[60, 80)	[30, 60)	[0, 30)
C15	million m^3^/km^2^	+	>50	(30, 50]	(20, 30]	(10, 20]	[0, 10]
C16	%	±	[25, 30]	[20, 25)	[10, 20)	[5, 10)	[0, 5)
				(30, 40]	(40, 50]	(50, 60]	(60, 100]
C17	%	+	[90, 100]	[80, 90)	[60, 80)	[30, 60)	[0, 30)
C18	million	+	>15	(15, 12]	(12, 10]	(10, 5]	(5, 0]
C19	m^3^	−	[0, 15)	[15, 25)	[25, 45)	[45, 80)	>80
C20	-	+	[90, 100]	[80, 90)	[60, 80)	[30, 60)	[0, 30)
C21	-	+	>3	(2, 3]	(1, 2]	(0, 1]	0
C22	%	+	[90, 100]	[80, 90)	[60, 80)	[30, 60)	[0, 30)

**Table 4:** Weights of the multifunctional river assessment.

Criterion Layer	Weight			Criticality	Indicator Layer	Weight			Criticality
	AHP	Entropy Method	Comprehensive			AHP	Entropy Method	Comprehensive	
A1	0.2742	0.3279	0.3010	1	C1	0.0619	0.0907	0.0763	1
					C2	0.0838	0.0000	0.0419	14
					C3	0.0220	0.0422	0.0321	19
					C4	0.0417	0.0638	0.0528	5
					C5	0.0324	0.0428	0.0376	16
					C6	0.0324	0.0884	0.0604	3
A2	0.2938	0.2195	0.2566	2	C7	0.0543	0.0317	0.0430	13
					C8	0.0321	0.0382	0.0352	17
					C9	0.0208	0.0365	0.0286	20
					C10	0.0501	0.0505	0.0503	7
					C11	0.0684	0.0301	0.0492	9
					C12	0.0681	0.0325	0.0503	7
A3	0.2886	0.2031	0.2458	3	C13	0.1143	0.0285	0.0714	2
					C14	0.0572	0.0391	0.0481	11
					C15	0.0478	0.0491	0.0485	10
					C16	0.0276	0.0415	0.0346	18
					C17	0.0417	0.0448	0.0433	12
A4	0.1434	0.2945	0.1964	4	C18	0.0402	0.0744	0.0573	4
					C19	0.0489	0.0536	0.0512	6
					C20	0.0148	0.0346	0.0247	21
					C21	0.0247	0.0555	0.0401	15
					C22	0.0148	0.0314	0.0231	22

## Data Availability

Not applicable.
